# Dual-energy computed tomography to detect early pulmonary vascular changes in children with sickle cell disease: a pilot study

**DOI:** 10.3389/fped.2023.1221977

**Published:** 2023-08-30

**Authors:** Raphael Joye, Julie Wacker, Duy-Anh Nguyen, Anne-Lise Hachulla, Albane B. R. Maggio, Laurent Cimasoni, Frederic Lador, Marc Ansari, Maurice Beghetti

**Affiliations:** ^1^ Pediatric Cardiology Unit, Department of Woman, Child, and Adolescent Medicine, Geneva University Hospitals, Geneva, Switzerland; ^2^ Pulmonary Hypertension Program, Geneva University Hospital, Geneva, Switzerland; ^3^ Division of Radiology, Diagnostic Department, Geneva University Hospitals, Geneva, Switzerland; ^4^ Health and Movement Consultation, Department of Woman, Child, and Adolescent Medicine, Geneva University Hospitals, Geneva, Switzerland; ^5^ Division of Pediatric Oncology and Hematology, Department of Women, Child and Adolescent, University Geneva Hospitals, Geneva, Switzerland; ^6^ Division of Pneumology, Geneva University Hospitals, Geneva, Switzerland; ^7^ Cansearch Research Platform for Pediatric Oncology and Hematology, Faculty of Medicine, Department of Pediatrics, Gynecology and Obstetrics, University of Geneva, Geneva, Switzerland

**Keywords:** pulmonary hypertension, sickle cell disease, dual-energy computed tomography, screening, children

## Abstract

**Introduction:**

Pulmonary hypertension (PH) is a rare but fatal complication of sickle cell disease (SCD) that is possibly reversible if treated early. Dual-energy computed tomography (DECT) is a valuable tool for diagnosing PH. We attempted to determine if DECT can detect early signs of PH in children with SCD.

**Methods:**

This prospective observational pilot study was conducted at the Geneva University Hospitals and was approved by the local human ethics committee (CCER 2019-01975). A written informed consent was obtained from the patients and/or their legal guardian. Eight children (consisting of five girls and three boys) with homozygous SCD were included in the study. They underwent full cardiological workup using transthoracic echocardiography (TTE) and cardiopulmonary exercise test (CPET), as well as DECT.

**Results:**

The median age of the children was 11 years old (range 8–12). All patients exhibited a normal biventricular systo-diastolic function using the TTE. The median tricuspid regurgitant jet velocity value was 2.24 m/s (range 1.96–2.98). Four children were found to have signs of vasculopathy detected on DECT. Of them, two had abnormal screening test results. They both had an increased VE/VCO_2_ slope during CPET and an increased TVR of >2.5 m/s on TTE.

**Conclusion:**

DECT is capable of identifying early signs of pulmonary vascular disease in children with SCD. Further studies are needed to understand the correlation between DECT abnormalities and hemodynamic pulmonary circulation better.

## Introduction

Sickle cell disease (SCD) is an autosomal recessive disorder associated with a single point mutation in the *β*-globin gene leading to hemoglobin polymerization, erythrocyte sickling, and hemolysis under hypoxia or acidosis. The inheritance of homozygous mutant hemoglobin S constitutes the most common form of SCD and is typically associated with severe hemolytic anemia and increased risk of thrombosis (1). Precapillary pulmonary hypertension (PH) has been identified as a fatal complication of SCD (2). In adults and children older than 3 months of age, PH is defined as an increased mean pulmonary arterial pressure (mPAP) of >20 mmHg that is measured by performing right heart catheterization (RHC). Furthermore, it is recommended to identify precapillary PH with an indexed pulmonary vascular resistance (PVR) of ≥3 WU/m^2^ and a pulmonary arterial wedge pressure of ≤15 mmHg. Based on clinical criteria, PH is classified into five groups, with SCD belonging to group 5 (3, 4). Chronic hemolysis leading to nitric oxide depletion and endothelial dysfunction, combined with chronic thromboembolic events, appears to be the main component of pulmonary vasculopathy (5). Furthermore, in adults with SCD, the estimated prevalence of PH with a tricuspid regurgitant jet velocity (TRV) of ≥2.5 m/s using transthoracic echocardiography (TTE) reaches 40% (2, 6). In contrast, hemodynamic studies reported a lower prevalence of PH, ranging from 6% to 10.4%, as confirmed by RHC (2, 7, 8). The overall mortality rate of PH in the SCD population for over an 18-month follow-up period has been reported to be as high as 5.3% (6). Therefore, PH significantly contributes to the increased mortality rate among patients with SCD. Indeed, TRV, mPAP, PVR, transpulmonary gradient, and N-terminal pro-brain natriuretic peptide (NT-proBNP) have been associated with up to a 14-fold increased risk of mortality in these patients (8, 9).

Among the children with SCD, while RHC remains the gold standard for diagnosing PH, a reliable non-invasive test is still lacking, and a combination of TTE and NT-proBNP is recommended when performing the screening test based on the studies on the adult population (10). The estimated prevalence of TRV ≥ 2.5 m/s is approximately 30%, nearly the same as that reported in the adult population (11). However, TRV lacks sensitivity and specificity to diagnose PH in children and young adults (11), and an increased TRV has not been associated with death in this population (12). Furthermore, PH in SCD patients was hypothetically a progressive condition from childhood to adulthood with potential reversibility (11).

Dual-energy computed tomography (DECT) is an imaging technology that provides a combined morphological analysis and functional information on pulmonary perfusion. Based on the attenuation properties of iodine, DECT provides pulmonary blood volume maps and quantifies the iodine concentration in the parenchyma, thus allowing simultaneous analysis of the gray-scale vasculature and color-scale parenchymal perfusion (13). In infants with chronic lung diseases and/or PH, DECT exhibited a similar efficacy to ventilation/perfusion scintigraphy for lung perfusion assessment (14). Moreover, it had been demonstrated that DECT is a valuable tool for diagnosing PH, regardless of the underlying mechanism or the age of the patient (13, 15). In adults with suspected PH, it had recently been shown that pulmonary blood volume obtained through DECT was independently correlated with systolic pulmonary arterial pressure estimated by TTE (16). Due to the lack of screening tools to diagnose PH in children with SCD, we hypothesized that early signs of pulmonary vasculopathy can possibly be detected by using DECT.

## Methods

This prospective observational pilot study was conducted at the Geneva University Hospitals and was approved by the local human ethics committee (CCER 2019-01975). A written informed consent was obtained from the patients and/or their legal guardian.

Eight children (consisting of five girls and three boys, aged 8–18 years), with homozygous SCD, were included in the study between 1 June and 31 December 2020. The exclusion criteria were the presence of any pulmonary disease other than PH, allergy to iodine, and pregnancy. Medical history, physical exam, blood test, and TTE were performed during a visit to a cardiology outpatient clinic. The patient then underwent cardiopulmonary exercise test (CPET) and DECT.

CPET is a recommended additional test in the diagnostic algorithm for pediatric PH (3). It is performed on a pediatric cycle ergometer, and oxygen consumption is measured by direct gas analysis (MetaLyzer® 3B/II, Cortex, Leipzig, Germany). MetaLyzer® 3B is a breath-by-breath device, whereas MetaLyzer® II uses the mixing chamber technique. Blood gases are obtained by capillary blood sampling of the earlobe or fingertip, before the start of the test and once the test is finished. Data are analyzed by using ABRM on the manufacturer software (MetaSoft®, Cortex, Leipzig, Germany).

DECT was performed on a second-generation dual-source 128-slice multiple-detector CT system (Somatom Definition Flash, Siemens Healthcare, Forchheim, Germany). Iodixanol contrast (Visipaque: 270 mg of iodine/ml, GE Healthcare, Switzerland) was administered at 2 ml/kg for patients weighing <40 kg and at 80 ml for patients weighing >40 kg. The DECT patterns were encoded and described by AH according to the classification published by Giordano et al. (17) (see Supplementary Material).

Importantly, the cardiological workup, the CPET, and the DCET were all performed at least 1 month after the last acute pulmonary injury, such as acute chest syndrome or pneumonia.

## Results

The median age of the participants was 11 years old (range 8–12). History of previous vaso-occlusive crises was ubiquitous. However, only three participants had experienced acute chest syndrome. The median hemoglobin level was 75 g/L (range 51–100), and six patients were on hydroxyurea therapy. Hemolysis markers were elevated in all patients with a median lactate dehydrogenase value of 500 U/L (range 332–675) and a median total bilirubin value of 45 μmol/L (range 18–107). Of note, none of the participants had any history of liver injury. Demographic, clinical, and biological characteristics of the patients are shown in Supplementary Table S1.

All eight patients underwent a full cardiological workup at the time of inclusion. One patient was on World Health Organization functional class (WHO-FC) III, three patients were on WHO-FC II, and the remaining were reported to have no symptoms. Cardiac biomarkers were within the normal range in all patients, with a median troponin value of 3 ng/L (range 3–9) and a median NT-proBNP value of 40 ng/L (range 13–58). The TTE exhibited a normal anatomy and normal biventricular systolic function in all patients. The median TRV value was 2.24 m/sec (range 1.96–2.98) with two patients having had a TRV of ≥2.5 m/s. No other indirect signs of PH were reported, with normal pulmonary artery acceleration time (median 110 ms, range 100–160) and normal eccentricity index. The left ventricle was mildly dilated in four patients. Of these patients, the median end-diastolic left ventricular (LV) diameter was 5.1 cm (range 4.7–5.4). LV diastolic function was normal in all patients, with median ratio of peak early to late mitral inflow velocities (E/A) of 1.8 (range 1.6–2.2) and median ratio of the peak early mitral inflow velocity over the early diastolic mitral annular velocity (E/e’) of 5.9 (range 2.9–7.9). CPET was performed in seven patients. Cardiorespiratory fitness was decreased [with median peak oxygen uptake (VO_2_) value of 24 ml/kg/min], with only one patient reaching a VO_2_ above 75% of the predicted value. Two patients had a mild ventilation/perfusion mismatch during exercise with a minute ventilation/carbon dioxide production (VE/VCO_2_) slope slightly elevated (>35).

**Figure 1:**
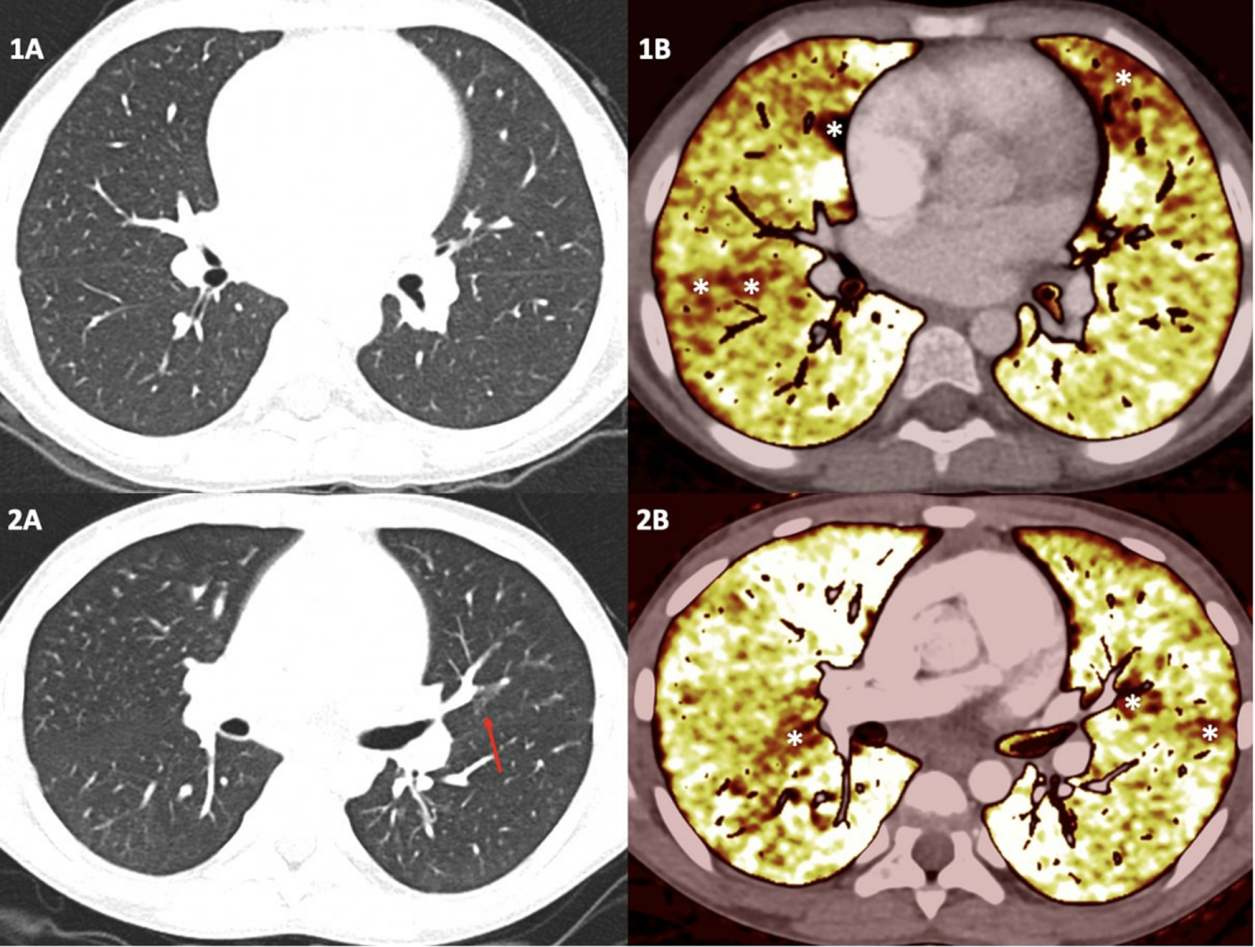
Dual-energy computed tomography results in two children with sickle cell disease. The top panel illustrates the case of an 8-year-old girl. No visible pulmonary artery emboli or parenchymal abnormalities on standard computed tomography pulmonary angiogram are seen (**1A**), but some patchy perfusion defect on the perfusion maps is depicted (**1B**) corresponding of a distal vascular disease. The bottom panel shows the case of an 11-year-old boy. No visible pulmonary artery emboli are detected. However, only a unique nodular ground-glass opacity on standard computed tomography pulmonary angiogram is seen (red arrow) (**2A**), and some patchy perfusion defects on the perfusion maps is depicted (**2B**).

DECT was performed in all patients. The median effective radiation dose was 1.44 mSv (range 0.82–1.68). These doses were comparable with other studies with a dual-energy protocol (15) or with a single-energy protocol (18). Radiation doses per patient are shown in Supplementary Table S2. Pathologic patterns were found in four of patients: two with nodular ground-glass opacities (GGO) associated with patchy patterns of abnormal perfusion, one with GGO and sequelae of infarction, and one with mosaic lung pattern (Figure 1). Among the four patients with pulmonary vasculopathy detected on DECT, only two had abnormal screening test: both had an increased VE/VCO_2_ slope during CPET and an increased TRV on TTE. The remaining two patients with pathological DECT had no other signs of PH during the cardiological workup. Finally, only two of the patients with abnormal findings detected on DECT had a history of acute chest syndrome. TTE and DECT findings are summarized in Table 1.

**Table 1:** Transthoracic echocardiography and dual-energy computed tomography findings.

	Transthoracic echocardiography						Dual-energy computed tomography
	Left ventricular end-diastolic diameter in cm	Left ventricular ejection fraction (%)	Left ventricular E/A	Left ventricular E/e’	Tricuspid regurgitant jet velocity in m/s	Pulmonary artery acceleration time in ms	
Patient 1	4	65	1.6	5	2.98	120	Nodular ground-glass opacities and patchy perfusion abnormalities
Patient 2	5.1	63	1.8	2.9	2.27	130	Nodular ground-glass opacities and sequelae of infarction
Patient 3	4.7	58	2	6.7	2.08	160	Nodular ground-glass opacities and patchy perfusion abnormalities
Patient 4	4.1	60	2.2	5.3	1.96	100	Normal
Patient 5	4.7	67	2.2	5.9	2.25	110	Normal
Patient 6	5.4	59	1.9	7.9	2.61	150	Mosaic lung pattern
Patient 7	5.4	63	1.8	6.1	2.13	110	Normal
Patient 8	4.8	57	1.7	6.3	2.24	110	Normal

E/A, ratio of peak early (E) to late (A) mitral inflow velocities; E/e’, ratio of the peak early mitral inflow velocity (E) over the early diastolic mitral annular velocity (e’).

## Discussion

This pilot study is the first to describe the use of DECT in children with SCD. We found that in eight children with a low probability of PH after the screening test performed that is recommended by the American Thoracic Society (10), half of them had abnormalities seen on DECT that were linked to pulmonary vasculopathy. Segmental or patchy perfusion defects noticed on the iodine maps of DECT were shown to correlate with PVR and also possibly help identify GGO of a vascular origin in patients with group 1 and group 5 PH (13, 19). Furthermore, mosaic lung patterns, patchy perfusion defects, and sequelae of infarction are well known signs of PH associated with chronic thromboembolic disease even without visible emboli on a standard computed tomography pulmonary angiogram (20). PH related to SCD seems to be a progressive condition that begins in childhood (11). Pulmonary vascular disease in patients with SCD is multifactorial and involves complex mechanisms such as chronic hemolysis, chronic thromboembolic events, and elevated post-capillary pressure due to LV diastolic dysfunction (21). Delayed diagnosis of PH in patients with SCD is one of the major causes of mortality. Therefore, early detection of pulmonary vascular lesions is of paramount importance to identify patients at risk of developing PH, in order to introduce specific therapies early in the course of the disease.

This study has some limitations. First, this is a pilot study with only a small number of patients. The small population size and the absence of a control group did not allow us to statistically measure associations. Therefore, inter-operator variability analysis is also lacking. Moreover, RHC was not performed in patients with abnormalities detected on DECT. It is to take note that DECT findings, such as GGO, are not specific to diagnose PH and can be found in viral infections. We tried to reduce this potential bias by including patients with no history of recent acute pulmonary injury or chronic lung disease. Therefore, further hemodynamic studies with catheterization values at rest and during exercise could offer a better understanding of the correlation between these early pulmonary vascular changes and the hemodynamic profile of the patients. Furthermore, studies with a longer follow-up might also be of interest to determine the correlation between early DECT findings and risk for future development of PH.

In conclusion, DECT has enough capability of identifying early signs of pulmonary vascular disease in a subset of patients with SCD. The place and timing of using DECT in the PH screening protocols and its correlation with hemodynamic parameters warrant further research.

## Data Availability

The original contributions presented in the study are included in the article/Supplementary Material, further inquiries can be directed to the corresponding author.
