# Supplementary material for: Dual-energy computed tomography to detect early pulmonary vascular changes in children with sickle cell disease: a pilot study
Source: Front Pediatr. 2023 Aug 30;11:1221977. doi: 10.3389/fped.2023.1221977 (PMC10498276; doi:10.3389/fped.2023.1221977)
Supplement: Supplementary file 1 [file Datasheet1.pdf]

Supplemental Digital Content

Classification of lesions on dual-energy computed tomography

A. <u>Image quality control</u>
    a. Diagnostic lung images
        Breathing artefacts
        - 0: no artifacts
        - 1: mild
        - 2: moderate
        - 3: no diagnostic
    b. Diagnostic mediastinal images
        i. Level of attenuation within the pulmonary trunk
        ii. Level of attenuation within the right pulmonary artery
        iii. Level of attenuation within the left pulmonary artery
    c. Perfusion images
        i. Beam-hardening artefacts due to contrast medium: 0-3
        ii. Cardiogenic artefacts: 0-3

B. <u>Diagnostic images</u>
    a. Patient-based analysis
        i. Lung abnormalities: Absence vs. Presence
    b. Segment-based analysis
        i. Lung segments with abnormal computed tomography features
        ii. Patterns of abnormal computed tomography features:
            1. Acute pulmonary embolism vs. Chronic pulmonary embolism
            2. Mosaic attenuation vs. Mosaic perfusion
            3. Diffuse ground glass vs. Nodular ground-glass opacities
            4. Neovascularity: tiny and serpiginous intrapulmonary vessels
            5. Infiltrates: Infection vs. Sequelae of infarction
            6. Fibrosis

C. <u>Perfusion analysis</u>
    a. Patient-based analysis
        i. Normal perfusion vs. Abnormal perfusion
    b. Segment-based analysis
        i. Number of segments with perfusion abnormalities
        ii. Patterns of perfusion abnormalities:
            a. Triangular
            b. Patchy
            c. Diffuse

	Sex	Age in years	WHO functional class	History of acute chest syndrome (number of episodes)	Laboratory data				
					Hemoglobin in g/l	Lactate deshydrogenase in U/l	Total bilirubin in mcmol/l	Troponin in ng/l	NT-pro BNP in ng/l
**Patient 1**	Female	8	II	Yes (1)	83	419	45	3	45
**Patient 2**	Male	11	II	No	77	500	17	3	33
**Patient 3**	Male	11	II	Yes (1)	75	461	23	4	40
**Patient 4**	Female	9	No symptoms	No	73	547	47	3	77
**Patient 5**	Female	8	No symptoms	No	51	675	48	7	33
**Patient 6**	Female	12	III	No	78	544	26	9	49
**Patient 7**	Female	12	No symptoms	No	73	576	117	3	13
**Patient 8**	Male	11	No symptoms	Yes (1)	100	332	18	3	58

Table 1. Patients' clinical and biological characteristics.

WHO: World Health Organization; NT-proBNP: N-terminal pro-brain natriuretic peptide

	**Effective radiation dose in mSv**
**Patient 1**	0.82
**Patient 2**	1.44
**Patient 3**	1.29
**Patient 4**	0.86
**Patient 5**	1.68
**Patient 6**	1.39
**Patient 7**	1.64
**Patient 8**	1.54

Table 2 : Radiation doses detail.
mSv : millisievert
